# Dissecting the Biological Relevance and Clinical Impact of lncRNA MIAT in Multiple Myeloma

**DOI:** 10.3390/cancers13215518

**Published:** 2021-11-03

**Authors:** Katia Todoerti, Domenica Ronchetti, Noemi Puccio, Ilaria Silvestris, Vanessa Favasuli, Nicola Amodio, Massimo Gentile, Fortunato Morabito, Antonino Neri, Elisa Taiana

**Affiliations:** 1 Department of Oncology and Hemato-Oncology, University of Milan, 20122 Milan, Italy; katia.todoerti@studenti.unimi.it (K.T.); domenica.ronchetti@unimi.it (D.R.); noemi.puccio@studenti.unimi.it (N.P.); ilaria.silvestris@unimi.it (I.S.); vanessa.favasuli@unimi.it (V.F.); elisa.taiana@unimi.it (E.T.); 2 Hematology, Fondazione Cà Granda IRCCS Policlinico, 20122 Milan, Italy; 3 Department of Experimental and Clinical Medicine, Magna Graecia University of Catanzaro, 88100 Catanzaro, Italy; nikamodio@hotmail.com; 4 Hematology Unit, “Annunziata” Hospital of Cosenza, 87100 Cosenza, Italy; massimogentile@virgilio.it; 5 Hematology and Bone Marrow Transplant Unit, Hemato-Oncology Department, Augusta Victoria Hospital, East Jerusalem 91191, Israel; fmorabito@avh.org; 6 Biotechnology Research Unit, Azienda Ospedaliera di Cosenza, 87100 Cosenza, Italy

**Keywords:** MIAT, multiple myeloma, bortezomib

## Abstract

**Simple Summary:**

The interest in the biological role and clinical impact of long non-coding RNAs (lncRNAs) in multiple myeloma (MM) is continuously expanding. Many studies, mainly involving solid tumors, have strongly suggested the MIAT oncogenic role; more recently, it has been suggested that MIAT may have a role in inducing Bortezomib resistance in MM. However, data concerning MIAT deregulation in MM are virtually absent. In this context, we investigated the expression pattern and the clinical relevance of the lncRNA MIAT in MM, taking advantage of the publicly available CoMMpass database. Our findings prompt further studies to elucidate better the significance of MIAT in MM.

**Abstract:**

The biological impact of long non-coding RNAs (lncRNAs) in multiple myeloma (MM) is becoming an essential aspect of the investigation, which may contribute to understanding the disease’s complex pathobiology, providing novel potential therapeutic targets. Herein, we investigated the expression pattern and the clinical relevance of the lncRNA MIAT in MM, taking advantage of the publicly available CoMMpass database. MIAT expression in MM is highly heterogeneous and significantly associated with specific molecular lesions frequently occurring in MM. Transcriptome analyses of MM PCs from patients included in the CoMMpass database indicated a potential involvement of MIAT in different signaling pathways and ribosome biogenesis and assembly. These findings suggest that MIAT deregulation may play a pathogenetic role in MM by affecting both proliferation pathways and, indirectly, the translational process. Although MIAT expression levels seem not to be significantly associated with clinical outcome in multivariate analyses, high MIAT expression levels are associated with bortezomib resistance, this suggesting that MIAT targeting could overcome drug resistance in MM. These findings strongly prompt for further studies investigating the significance of MIAT in MM.

## 1. Introduction

Multiple Myeloma (MM) is a malignant proliferation of bone marrow plasma cells (PCs) characterized by a broad clinical spectrum ranging from the pre-malignant condition called monoclonal gammopathy of undetermined significance (MGUS) to extra-medullary myeloma/plasma cell leukemia (PCL) [1]. MM accounts for approximately 10% of hematological malignancies, and despite the recent improvement in treatment and patient care, it remains an incurable disease [2]. MM is characterized by a profound genomic instability resulting in structural and numerical chromosomal aberrations, some of which, i.e., chromosomal translocations involving the immunoglobulin heavy chain (IGH) locus at chromosome 14q32 with an extensive array of putative proto-oncogenes, or trisomies involving odd chromosomes, are thought to represent early and specific events in myelomagenesis [3]. More recently, mutations affecting several genes, such as *KRAS*, *NRAS*, *TP53*, *BRAF*, *TRAF3*, *FAM46C*, and *DIS3*, have been identified and extensively characterized [4,5,6,7,8,9,10]. Overall, these events may have a profound impact on the biology and clinical outcome of the disease.

Over the past years, we and others have provided valuable information on coding and non-coding transcriptomic profiles associated with the main molecular types of MM. These studies have been of high relevance to understand altered molecular pathways in the disease and identify novel prognostic and predictive biomarkers and putative therapeutic targets [2,11,12]. LncRNAs represent more than half of the mammalian non-coding transcriptome and are involved in many biological processes, including cis or trans transcriptional regulation, organization of nuclear domains, and proteins or RNA molecules [13,14]. LncRNAs interact with DNA, RNA, or proteins and perform several non-mutually exclusive functions such as signals, decoy, guide, or scaffold [15]. Notably, lncRNAs are critical drivers of tumorigenesis by promoting all hallmarks of cancer [16].

The biological role and therapeutic potential of lncRNAs in MM are poorly defined [11,17]. Studies from our and other groups, based on microarray or RNA-Seq technology, contributed to providing a catalogue of lncRNAs in MM specifically deregulated in the context of the main genetic alterations and molecular subgroups and potentially relevant for the disease outcome [18,19,20]. In such a context, few lncRNAs have been functionally investigated in MM [11], including MALAT1 and NEAT1, both closely located at chromosome 11q and reported associated with other types of human cancer. We contributed to demonstrate that MALAT1 promotes cell survival by regulating the proteasome machinery and provided evidence of its druggability in vitro and in vivo [21]. NEAT1 represents an indispensable structural component of nuclear paraspeckles (PSs), a class of dynamic lncRNA-directed nuclear bodies, potentially involved in the nuclear sequestration of specific RNAs or proteins and stress responses [22]. We recently demonstrated that NEAT1 is up-regulated in MM [23] and its silencing antagonizes MM cell growth both in vitro and in vivo and affects the Homologous Recombination (HR) repair pathway, which may explain the synergistic impact with several drugs, including bortezomib (BTZ), carfilzomib, melphalan, and PARP inhibitors [24]. Overall, these findings suggest that MALAT1 and NEAT1, and more in general lncRNAs, could represent promising targets for novel anti-MM therapies.

In the context of our previous analyses concerning the transcription profiles of lncRNA in a limited panel of MM patients, we found myocardial infarction-associated transcript (MIAT) as significantly deregulated in MM patients except for those harboring the t(11;14) translocation [18]. Many studies, mainly involving solid tumors, have strongly suggested the MIAT oncogenic role [25,26]. MIAT plays an essential role in controlling cell proliferation, invasion, metastasis, and apoptosis through different mechanisms, including competitive endogenous RNA (ceRNA) or regulator of gene transcription and signaling pathways [25]. More recently, it has been suggested that MIAT may have a role in inducing BTZ resistance in MM, being found as a BTZ-inducible lncRNA and significantly increased in BTZ-resistant patients [27].

Prompted by these intriguing pieces of evidence, we took advantage of the genomic and transcriptomic data included in the MMRF CoMMpass dataset to dissect the impact of MIAT expression extensively in the context of the MM genomic landscape and transcriptome to elucidate better its role in MM.

## 2. Materials and Methods

### 2.1. Multi-Omics Data in CoMMpass Study

Multi-omics data about bone marrow MM samples at baseline (BM_1) were freely accessible from MMRF CoMMpass Study (https://research.themmrf.org/, accessed on 16 October 2020) including more than 1000 MM patients from several worldwide sites and retrieved from the Interim Analysis 15a (MMRF_CoMMpass_IA15a). Details about molecular and clinical data of the CoMMpass cohort selected for the present study are described in the Supplementary Materials.

### 2.2. Statistical and Survival Analyses

Wilcoxon rank-sum and Kruskal–Wallis tests were applied to assess differential expression patterns between two or multiple molecular groups. Dunn’s test was used for pairwise comparisons. Fisher’s exact test was applied to verify the association between genomic alterations in stratified MM cases. *p*-values were corrected using Benjamini–Hochberg (BH) method, and adjusted *p*-values < 0.05 were considered significant. Survival analyses were performed using survival and survminer packages in R Bioconductor (version 4.0.0). To provide a cut-point value that corresponds to the most significant relationship with survival and to stratify MM cases of CoMMpass cohort in high and low MIAT expression groups, the optimal cut-off for MIAT expression level was based on maximally selected rank statistics (max-stat) method. The global 767 MM dataset was half randomly split into train and test sets and the best max-stat cut-off in association to OS was chosen and validated in test and global datasets, as explained in Supplementary Materials.

### 2.3. Differential Expression Analysis on CoMMpass MM Cohort

A global dataset of 774 BM_1 MM cases was stratified according to RNA-seq MIAT expression levels. Further steps of analysis are described in Supplementary Materials. A Volcano plot was used in R to represent significantly up- or down-regulated transcripts.

### 2.4. Functional Enrichment Analysis on Differentially Expressed Protein-Coding Genes

Gene Set Enrichment Analysis (GSEA) was performed on the pre-ranked differentially expressed (DE) protein-coding gene lists based on the fold change (FC) values by computing 1000 permutations and using default analysis conditions. Further details about GSEA analysis are reported in Supplementary Materials.

## 3. Results

### 3.1. MIAT Expression in MM Patients

We have previously identified lncRNA MIAT in a proprietary MM dataset profiled by microarrays, as one of the most significantly down-regulated lncRNAs in patients specifically carrying the t(11;14) in comparison to all others [18]. Notably, when we compared the MIAT expression levels of the four healthy donors, 50 MM and 21 PCL cases included in our dataset, we observed a similar low median value in all the groups, although heterogeneous expression levels were evidenced in tumor samples, mainly in MM group (Supplementary Figure S1). These findings prompted us to investigate the MIAT expression pattern in a larger cohort of MM patients that could be more representative of the clinical and genomic heterogeneity of the disease. To this end, we evaluated the MIAT global expression levels in 774 BM-1 MM cases included in the CoMMpass dataset. According to the previous data, MIAT expression spanned a wide range of estimated values (0.13–762; median: 11.14) for transcripts per million (TPM), with more than two-thirds of the t(11;14) MM cases (113/148, 76%) showing MIAT expression level lower than the median value across the entire cohort (Supplementary Figure S2).

**Figure 1:**
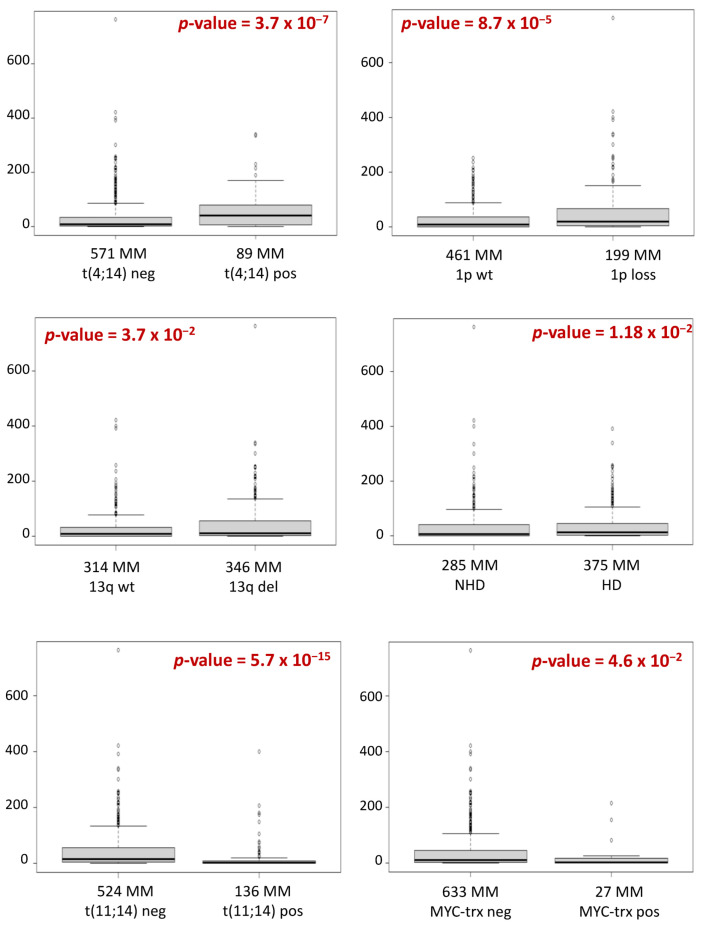
Box plots of MIAT expression level in 660 MM cases stratified according to the presence of t(4;14), del(1p), del(13q), hyperdiploidy (HD), t(11;14) or MYC translocations (MYC-trx). Differential expression was tested by Wilcoxon rank-sum test with continuity correction. *p*-values were corrected by BH adjustment.

To assess MIAT expression profiles in relation to major molecular aberrations in MM, we investigated 660 MM patients of the CoMMpass cohort for which expression, Non-Synonymous (NS) somatic mutation, and Copy Number Alterations (CNAs) data were available by RNA-sequencing (RNA-seq), Whole Exome Sequencing (WES) and next generation sequencing (NGS)-based FISH (FISH-WES), respectively (Supplementary Table S1). Significant higher MIAT expression levels were observed in MM patients carrying t(4;14), del(1p), del(13q), or the hyperdiploid (HD) status, whereas relevant lower expression levels were evidenced in t(11;14) or MYC-translocated cases (Figure 1). No significant differences in MIAT expression levels were observed in relation to t(6;14), MAF translocations, 1q gain, 17p/*TP53* deletion, or mutations affecting *DIS3*, *RAS/BRAF*, *TP53*, *FAM46C*, or *TRAF3* genes (Supplementary Figure S3).

### 3.2. Clinical Impact of MIAT Expression in CoMMpass Cohort

**Figure 2:**
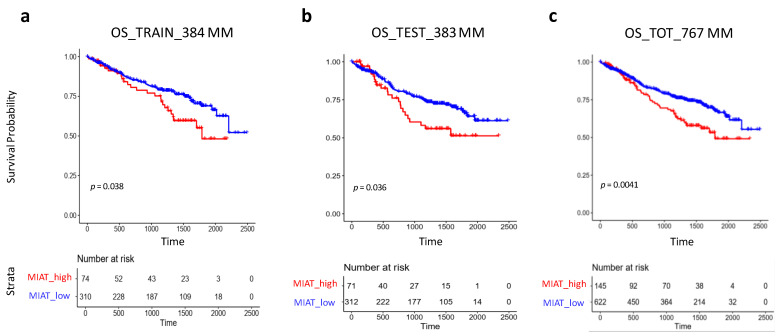
Kaplan–Meier survival curves in 384 MM train (**a**) and 383 MM test (**b**) sets that were randomly selected from total 767 MM dataset. MM cases were stratified in high and low MIAT expression groups, accordingly to max-stat cut-off identified in the train set with respect to Overall Survival (OS) data. (**c**) Kaplan–Meier survival curve based on the same threshold in 767 global dataset: OS median follow-up time: not reached for low group, 1789 days for high group; median follow-up time of the study 1429 days; Inter Quartile Range (IQR): [796; 1718]. Log-rank test *p*-values measuring the global difference between survival curves and number of samples at risk in each group across time are reported.

In order to investigate the relevance of MIAT expression levels in clinical outcome, we considered 767 MM patients with available clinical data. For this purpose, the entire dataset was half randomly split into two subsets (see Section 2). In details, a training set of 484 MM cases was used to define an estimated cut-point on MIAT expression level corresponding to the most significant relation with overall survival (OS), that was based on maximally selected log-rank statistics (max-stat cut-off) (Supplementary Figure S4 and Figure 2a), whereas a test set of 483 MM cases was used to validate the identified maxstat threshold. Notably, in both the training and test sets, a poorer clinical outcome was found in MM group expressing higher MIAT levels in relation to OS (Figure 2b); this finding was more significant if the global 767 MM dataset (145 high MIAT MMs compared to 622 low MIAT MMs) was considered (Figure 2c). Aiming to verify if the clinical response of MM cases stratified according to MIAT expression levels could significantly differ also in relation to PFS, we tested randomly split train and test pairs without observing any significant difference.

**Figure 3:**
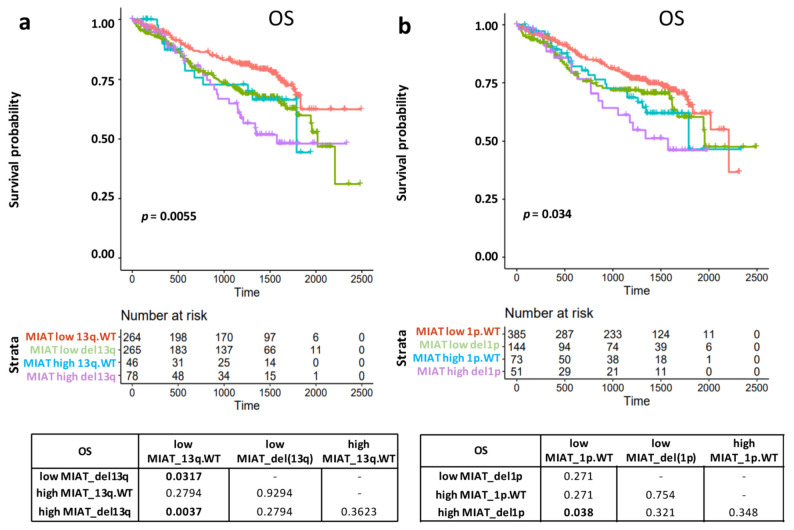
(**a**,**b**) Kaplan–Meier survival curves in 653 MM with expression, molecular and clinical data available. Log-rank test *p*-values measuring the global difference between survival curves and the number of samples at risk in each group across time are reported. Log-rank test *p*-values of pairwise comparisons are also reported; significant adjusted *p*-values by BH correction (<0.05) are in bold.

Based on previous evidence and the observed differential expression patterns according to main molecular alterations (Figure 1), we wanted to evaluate the possible impact on survival of MIAT expression level in combination with the other significantly associated molecular variables, like t(11;14), t(4;14), MYC-trx, del(13q)/RB1, del(1p)/CDKN2C or HD. Notably, the combination of higher MIAT expression level with the occurrence of 13q/RB1 or 1p/CDKN2C deletions was associated with the poorest survival rate in OS (Figure 3a,b). In contrast, the most favorable one was identified in the lower MIAT expression group respectively associated with a normal copy number (CN) state at 13q/RB1 (Figure 3a) (BH adj. Log-Rank *p*-value = 0.0037) or 1p/CDKN2C (BH adj. Log-Rank *p*-value = 0.038) (Figure 3b). No significant differences were detected for MIAT expression combined with t(11;14), t(4;14), MYC-trx, or HD condition.

To verify if high MIAT expression levels may represent an independent variable in predicting OS, we tested high MIAT expression condition and other main molecular or clinical features by Cox regression univariate analysis in 497 MM for which all information records were available. A significantly higher risk of death was observed for cases with higher MIAT expression level (Hazard Ratio, HR = 1.74, 95% CI 1.16–2.63, BH adj. *p*-value = 0.027), together with older age (equal or over 65 years) [28,29], ISS stage III, and distinct molecular variables such as del(13q)/RB1, del(1p)/CDKN2C, HD, and 1q gain/amplification alone or in combination with TP53 alterations (Supplementary Figure S5a). Conversely, ISS stage I cases showed a 69% death risk reduction. However, when all significant variables were tested in multivariate analysis, MIAT expression level lost its independence in predicting a poorer OS and only 1q gain/amplification in combination with TP53 alterations, older age, ISS stages III and I retained significance (Supplementary Figure S5b).

Finally, we investigated if the combination of high MIAT expression level with the occurrence of 13q/RB1 or 1p/CDKN2C deletions may represent an independent variable in predicting OS. A significantly higher risk of death was observed for cases with high MIAT expression level and del(13q) (Hazard Ratio, HR = 2.2, 95% CI 1.4–3.4, BH adj. *p*-value = 0.0029), and for patients with the combination high MIAT expression level and del(1p) (Hazard Ratio, HR = 2.95%, CI 1.1–3.6, BH adj. *p*-value = 0.035) (Supplementary Figure S6a–c). However, in multivariate analysis, also these combinations lost their independence in predicting a poorer OS (Supplementary Figure S6b–d).

### 3.3. Impact of MIAT Expression in Treatment Response

**Figure 4:**
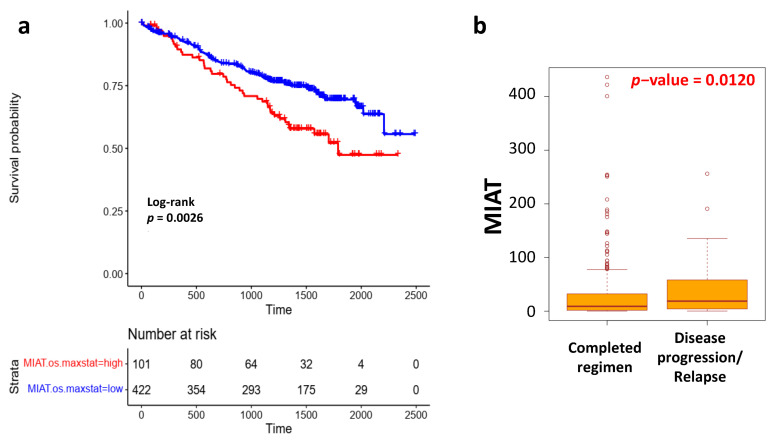
(**a**) Kaplan–Meier survival curve in 523 MM cases treated with bortezomib, bortezomib/IMIDs, or bortezomib/IMIDs/carfilzomib in high compared to low MIAT group, according to previously defined max-stat cut-off relative to OS. Log-rank test *p*-value is reported. (**b**) Box plots of MIAT expression level in 287 MM cases that reached a completed regimen in comparison to 69 MM patients who experienced disease progression/relapse after bortezomib treatment. Differential expression was tested by the Wilcoxon rank-sum test with continuity correction.

Then, we considered the impact of MIAT expression level in the context of the first-line treatment regimens in CoMMpass cohort (Supplementary Figure S7). Particularly, based on the suggested role of MIAT in molecular mechanisms of bortezomib resistance in MM disease [27], we assessed whether the correlation between OS and MIAT expression could be a hallmark of bortezomib-treated patients. To this purpose, we tested if the previously defined maxstat threshold could also discriminate survival of high and low MIAT MM cases in the subset of 523 MMs that were treated with bortezomib alone (142 MM), in combination with IMIDs (356 MM), or IMIDs and carlfizomib (25 MM). Our analysis demonstrated a significantly lower OS in the high MIAT cases compared to the low MIAT MM patients (Figure 4a). Additionally, in the context of bortezomib-based therapies, significantly higher MIAT levels were observed in 69 MM cases who experienced disease progression/relapse after bortezomib treatment alone or in combined regimens, compared to 287 MMs patients who completed those therapeutic regimens (Figure 4b).

On the other hand, no relevant differences in clinical outcome were observed in 157 patients who underwent different treatment regimens that did not include bortezomib (39 IMIDs, 5 carlfizomib, 113 IMIDs and carlfizomib) (Supplementary Figure S8).

### 3.4. Transcriptional Signature and Molecular Pathways Specifically Associated with MIAT Expression

In order to define the transcriptional pattern specifically associated with MIAT expression levels, we considered the 774 MM samples included in the RNA-seq CoMMpass dataset, stratifying them according to MIAT expression levels. Notably, more than 100-fold change (FC) in MIAT expression level was observed in top compared to bottom quartile (Supplementary Figure S9a).

At first, we investigated these two groups for the possible association with the main molecular lesions occurring in MM, showing a very significant positive correlation with t(4;14) (83% vs. 17%, BH adj. *p*-value = 8.50 × 10^−4^) and an inverse association with t(11;14) (16% vs. 84%, BH adj. *p*-value = 4.87 × 10^−2^), in MIAT fourth (168 MM) versus first (167 MM) quartile (Supplementary Table S2). In addition, a higher frequency of del(13q)/RB1 occurred in MM cases at higher compared to lower MIAT expression level (57% vs. 43%, BH adj. *p*-value= 4.87 × 10^−2^) (Supplementary Table S2).

Therefore, global expression profiles of annotated protein-coding genes (19.141 annotated protein coding genes by Ensembl Biomart) were compared in these two extreme quartiles. A list of 9813 differentially expressed (DE) protein-coding genes was obtained by limma analysis at a low stringency level (FDR 10% cut-off) (Supplementary Table S3), the majority of which resulted up-regulated (8834/9813, 90%) in patients with higher MIAT expression (Supplementary Figure S10).

**Figure 5:**
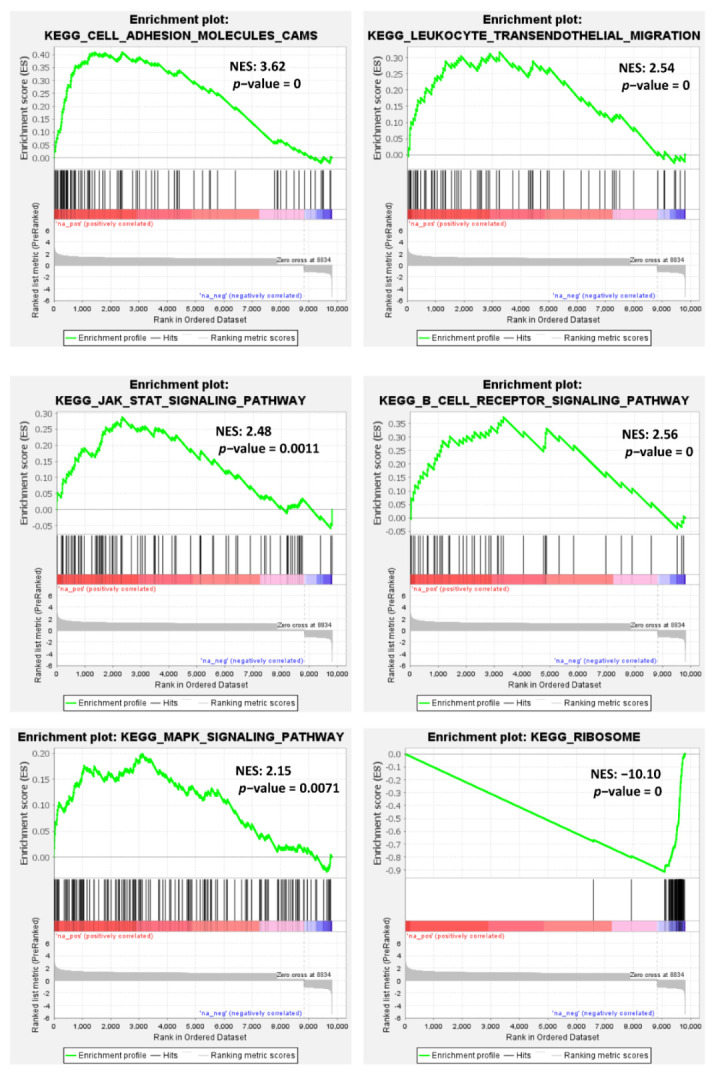
Enrichment plots of selected GSEA gene sets significantly modulated in MIAT IV versus I quartile. Normalized Enrichment Score (NES) and nominal *p*-value are reported for each plot.

To identify which molecular pathways could be modulated in relation to MIAT expression, we performed a Gene Set Enrichment Analysis (GSEA) on the list of DE coding genes ranked based on FC values, using different gene set collections. Notably, we found the positive modulation of transcripts involved in cell adhesion, cellular junctions and transendothelial migration, B cell receptor signaling, and other pathways, like those concerning JAK-STAT, MAPK, and TNFA-NFKB signaling (Supplementary Table S4, Figure 5). In addition, several gene sets associated with immune response or related to mitotic spindle or apoptosis and TP53 pathways were found up-regulated in MM cases with higher MIAT expression levels (Supplementary Table S4, Supplementary Figure S11). Conversely, genes involved in DNA repair or MYC target genes were found down-regulated in patients with higher MIAT expression (Supplementary Table S4, Supplementary Figure S11). To note, transcripts codifying for virtually all the protein constituents of the ribosome were also down-regulated in MM with higher MIAT expression levels (Figure 5 and Supplementary Table S5).

Notably, the significant association with specific molecular lesions, as previously described (Supplementary Table S2), showed a relevant impact on the transcriptional signature of MM cases stratified accordingly to MIAT expression. In particular, specific transcripts associated with the occurrence of major IGH chromosomal translocations, like those associated with the overexpression of the t(4;14)-target, WHSC1 gene, were found enriched in the comparison between MIAT extreme quartiles, whereas an opposite trend was evidenced for those genes related with CCND1-spiked expression in t(11;14) translocated cases (Supplementary Table S4).

To define a transcriptional signature more specifically associated with MIAT expression, avoiding the influence of the associated molecular alterations, we stratified MM samples according to MIAT expression level, without including t(4;14) or t(11;14) translocated cases (262 MM). Therefore, we compared 128 MM in upper versus 128 MM in lower quartile, thus maintaining approximately the same median expression level compared to previous extreme quartiles (Supplementary Figure S9b). A selection of 8353 differentially expressed protein coding genes was identified at the same stringency level (FDR 10%) by limma analysis, resulting mainly (92%) up-regulated at higher MIAT expression level and mostly overlapping with the previously identified list in the global CoMMpass cohort (6786 common transcripts, 81%). Accordingly, after a GSEA analysis on the 8353 DE protein-coding gene list, a vast number of enriched gene sets corresponded with previous GSEA results, also including those associated with main IgH translocations (Supplementary Table S4), thus underlining the high specificity of the identified MIAT transcriptional pattern independently of t(11;14) or t(4;14) presence.

## 4. Discussion

Despite accumulating evidence on transcriptional patterns and functional roles of lncRNAs in MM, their impact on MM pathobiology remains to be fully elucidated. In this study, we took advantage of the MMRF CoMMpass dataset and focused on MIAT, to shed light on the clinical relevance of its expression in the context of the MM genomic landscape. In addition, transcriptomic data were used to elucidate MIAT role in MM better.

MIAT shows a wide range of expression in MM PCs. Interestingly, this heterogeneity is significantly associated with specific molecular lesions frequently occurring in MM. In detail, we confirmed our previous data showing the lowest MIAT expression levels in MM carrying t(11;14) [18]. Conversely, higher MIAT expression levels were significantly associated with the presence of t(4;14) translocation, hyperdiploidy, 1p or 13q deletions. Notably, we found significantly lower MIAT expression levels in MYC-translocated patients, and, accordingly, we found a significant enrichment of MYC targets in MM cases expressing MIAT at lower levels, thus suggesting a possible relationship between MIAT and MYC in MM. Studies in solid tumors [30,31,32] and other pathological conditions [33,34] have already described an interplay between MIAT and MYC family genes based on different regulation mechanisms, suggesting that this relationship may depend on the cellular context. Further studies are needed to clarify the relationship in MM.

Data concerning the clinical relevance of MIAT deregulation in MM had reported high MIAT expression associated with worse prognosis in terms of both OS and PFS [27]. Our study performed in a larger and representative cohort of patients pointed out that higher MIAT expression levels were associated with poorer OS but not PFS. Furthermore, this alteration lost independent predictive power when tested with the other clinical and prognostic molecular variables used to foresee the clinical outcome. Moreover, we investigated if MIAT expression could acquire clinical relevance depending on the co-occurrence of other genetic lesions. In particular, we detected worse OS in patients with higher MIAT expression levels associated with del13q or del1p. However, when tested in multivariate analysis, even these combinations lost their independence in predicting poorer OS compared to other variables, as 1q gain/amplification alone or combined with *TP53* alterations, age equal to or over 65 years, and ISS stages III and I. Overall, our present data suggest that MIAT expression levels cannot be considered an independent prognostic marker in MM.

Recently, a clinical impact for MIAT has been suggested in inducing BTZ resistance in MM, being found as a BTZ-inducible lncRNA and significantly increased in BTZ-resistant patients [27]. Accordingly, MIAT silencing by shRNA lentivirus infection increased BTZ sensitivity in vitro by negatively regulating miR-29b, leading to control over the expression of miR-29b target genes. [27]. In line with this evidence, our data on patients treated with BTZ, alone or in combination with IMIDS or eventually followed by carfilzomib treatment, showed a significantly poorer outcome in OS for those cases at higher compared to lower MIAT expression levels. Additionally, significantly higher MIAT expression levels were observed in cases who experienced disease progression/relapse after BTZ treatment alone or in combined regimens compared to those that reached a completed regimen. Our results reinforce previous observations and suggest that MIAT druggability could overcome BTZ resistance in MM. In these regards, the use of the novel class of antisense oligonucleotides, such as locked nucleic acid (LNA) GapmeRs, may represent a valid tool for MIAT silencing. Indeed, this approach has been used in vitro [35] and in animal models [21,24,36]. In addition, their application was extended to clinical trials and to the treatment of some genetic diseases [37].

Despite that the MIAT role in the development of several human cancers has been acknowledged, its specific molecular mechanisms remain to be fully elucidated. Our transcriptomic analyses in MM unveiled molecular pathways and putative biological effects associated with MIAT modulation in primary tumors. Notably, high MIAT expression levels were associated with the positive modulation of transcripts involved in cell adhesion, cellular junctions, and transendothelial migration. This is not surprising as inhibition of cell migration and invasion upon MIAT depletion have been reported in various solid tumors [25,26]. Besides, dysregulation of MIAT has been associated with abnormal activity of numerous cancer-related signaling pathways such as B cell receptor, JAK-STAT, MAPK, and TNFA-NFKB signaling, indicating its role in the regulation of these signaling pathways. Therefore, MIAT can affect the carcinogenic process through different routes. Strikingly, high MIAT expression levels were associated with a dramatic reduction of ribosomal proteins. Concerning this aspect, the information in literature is very limited: to the best of knowledge, the involvement of lncRNAs in ribosome biogenesis has already been described only in breast cancer cells for lncRNA ZFAS1, whose expression is strongly correlated with that of a number of mRNAs encoding ribosomal proteins involved in ribosome biogenesis, and its abundance also increases upon induced ribosome biogenesis [38]. Based on this evidence, it would be of great interest to further investigate whether MIAT could interfere with the crucial ribosome biogenesis program and activity in MM, thus affecting translation not directly but instead regulating ribosome production and assembly.

## 5. Conclusions

Our results indicate that MIAT deregulation may play a pathogenetic role in MM by affecting different signaling pathways and, at the same time, influencing translation by regulating ribosome biogenesis and assembly. However, our present data indicate that MIAT expression levels are not significant predictive markers of clinical outcome in multivariate analyses but are involved in resistance to the proteasome inhibitors currently used in MM. Taken together, these findings strongly support the need of future investigations for the understanding of the mechanisms of deregulation and the biological role and activity of MIAT in MM.

## Data Availability

MMRF CoMMpass data are available at https://research.themmrf.org/ (accessed on 16 October 2020) and retrieved from the Interim Analysis 15a (MMRF_CoMMpass_IA15a).

## Supplementary Materials

The following are available online at https://www.mdpi.com/article/10.3390/cancers13215518/s1, Supplementary Materials. Supplementary Figure S1: Box plots of MIAT expression level in healthy donors (N) and PC dyscrasias proprietary dataset. Total RNA samples from highly purified bone marrow CD138+ plasma cells were profiled by Gene 2.0 ST array. Kruskal–Wallis test was applied to compare groups. Supplementary Figure S2: Box plot of MIAT expression level in main IgH translocation groups in 774 cases of the CoMMpass cohort. Kruskal–Wallis test was applied to compare groups. The significant pairwise comparison performed by the Dunn test are marked in bold red in the table. Supplementary Figure S3: Box plots of MIAT expression level in 660 MM cases stratified according to the presence of t(6;14), MAF translocations, 1qgain, del(17p)/TP53 or the occurrence of NS somatic mutations in *DIS3*, *RAS*/*BRAF*, *TP53*, *FAM46C*, or *TRAF3* genes. Differential expression was tested by Wilcoxon rank-sum test with continuity correction. *p*-values were corrected by BH adjustment. Supplementary Figure S4: (a) Determination of the optimal cut-point of MIAT expression level based on maximally selected rank statistics on 384 MM train set, randomly selected from 767 MM global dataset. A minimal proportion of 10% observable samples was set for each group. Samples with lower or equal value as cutpoint were classified in low, otherwise samples greater than cutpoint were classified in high group. Supplementary Figure S5: (a) Results of Cox regression univariate analysis using OS data on MIAT expression groups, age equal to or greater than 65 years, ISS subgroups and main molecular alterations in 497 BM-1 MM cases for which all data were available. Number (N) of positive cases is indicated for each variable. Hazard Ratio, 95% Confidence Interval and Log-rank *p*-value are reported for each variable. In bold red are depicted all significant variables after BH correction. (b) Forest plot of Cox regression multivariate analysis considering all features with adjusted *p*-value < 0.05 in univariate analysis with regards to OS in 497 BM-1 MM cases. Hazard Ratio, 95% Confidence Interval and Log-rank *p*-value are indicated in the plot for each variable. Significant *p*-value: * ≤0.05; ** ≤0.01; *** ≤0.001; **** ≤0.0001. Supplementary Figure S6: (a) Results of Cox regression univariate analysis using OS data on MIAT expression plus del13q groups, age equal to or greater than 65 years, ISS subgroups and main molecular alterations in 497 BM-1 MM cases for which all data were available. (b) Forest plot of Cox regression multivariate analysis considering all features with adjusted *p*-value < 0.05 in univariate analysis with regards to OS in 497 BM-1 MM cases. (c) Results of Cox regression univariate analysis using OS data on MIAT expression plus del1p groups, age equal to or greater than 65 years, ISS subgroups and main molecular alterations in 497 BM-1 MM cases for which all data were available. (d) Forest plot of Cox regression multivariate analysis considering all features with adjusted *p*-value < 0.05 in univariate analysis with regards to OS in 497 BM-1 MM cases. Hazard Ratio, 95% Confidence Interval and Log-rank *p*-value are indicated in the plot for each variable. Significant *p*-value: * ≤0.05; ** ≤0.01; *** ≤0.001; **** ≤0.0001. Supplementary Figure S7: Number of MM cases in each group of first-line therapy. Among 680 total MM cases with RNA-seq expression, clinical and therapy data, the ones with high MIAT expression levels according to max-stat cut-off with respect to OS are also indicated. Supplementary Figure S8: Kaplan–Meier survival curve in OS for 157 MM patients who underwent other regimens not including bortezomib. Samples were stratified in each dataset according to MIAT expression levels on the basis of previous OS maxstat threshold. Log-rank test *p*-value measuring the global difference between survival curves and number of samples at risk in each group across time are reported. Supplementary Figure S9: (a) MIAT expression levels in 774 MM of CoMMpass cohort according to quartiles. (b) MIAT expression levels in 512 MM dataset according to quartiles. Red boxes indicate the first and last quartiles in both datasets, the black one the discarded 262 MM cases carrying t(11;14) or t(4;14). Supplementary Figure S10: Volcano plot of 9813 significant DE protein coding transcripts between MIAT extreme quartiles. Up- and down-regulated transcripts with at least 2 FC absolute values are highlighted in red and blue, respectively. Supplementary Figure S11: Enrichment plots of selected GSEA gene sets significantly up- and down-regulated in MIAT IV versus I quartile. Normalized Enrichment Score (NES) and nominal *p*-value are reported for each plot. Supplementary Table S1: Number and relative frequency of main IgH translocations (trx), copy number alterations (CNAs), and non-synonymous (NS) somatic mutations, in 660 BM-1 MM cases of MMRF_CoMMpass_IA15a cohort with available data about MIAT expression by RNA sequencing (RNA-seq), IgH trx by RNA-seq, NS somatic mutations by Whole Exome sequencing (WES) and CNAs by next generation sequencing (NGS)-based FISH (FISH-WES). Supplementary Table S2: Results of Fisher’s exact test measuring the correlation between MIAT expression level and main IgH translocations (trx), copy number alterations (CNAs) and non-synonymous (NS) somatic mutations in recurrently mutated genes in CoMMpass cohort. del(13q) refers to 13q14, 13q34 cytobands or RB1 locus; 1q gain to 1q21 cytoband; del(1p) to 1p22 or CDKN2C locus; del(17p) to 17p13 or TP53 locus; HD to hyperdiploid condition. We used a dataset of 335 MMs stratified in the two extreme quartiles and with all available data (168 MM in the first quartile, 167 MM in the last quartile). Each set of *p*-values was adjusted by Benjamini–Hochberg (BH) method. Significant adjusted *p*-values (<0.05) were in bold. Supplementary Table S3: List of significant differentially expressed protein coding genes between MIAT extreme quartiles, by limma analysis at FDR 10% cut-off. 8834 up- and 979 down-regulated transcripts in quartile IV vs. I are respectively ordered according to adjusted *p*-value. Fold change (FC), *p*-value, and B statistics parameters are reported for each gene. Supplementary Table S4: List of significant gene sets enriched in last versus first MIAT quartile (194 MM in each group) by GSEA analysis. Up- and down-regulated gene sets of Kegg, Hallmark, c2 gene perturbations (c2gp) collections (version 7.2) are ordered according to nominal *p*-value and Normalized Enrichment Score (NES). C2gp gene sets were filtered on “Myeloma” term. * indicates gene sets that were enriched also by GSEA analysis on CoMMpass cohort excluding t(11;14) or t(4;14) MM cases. Supplementary Table S5: KEGG RIBOSOME gene set negatively associated with MIAT IV Quartile compared to MIAT I Quartile in CoMMpass database. Core Enrichment genes are shown in bold.
