# Supplementary material for: Dissecting the Biological Relevance and Clinical Impact of lncRNA MIAT in Multiple Myeloma
Source: Cancers (Basel). 2021 Nov 3;13(21):5518. doi: 10.3390/cancers13215518 (PMC8583301; doi:10.3390/cancers13215518)
Supplement: Supplementary file 1 [file cancers-13-05518-s001.zip › cancers-1421739-SI.pdf]

cancers

MDPI

Supplementary Methods

1.1. Multi-Omics Data in CoMMpass Study

Multi-omics data about bone marrow MM samples at baseline (BM_1) were freely available from MMRF CoMMpass Study (https://research.themmrf.org/ accessed on 16 October 2020) and obtained from the Interim Analysis 15a (MMRF_CoMMpass_IA15a).

Transcript per Million (TPM) reads values of the MIAT transcript were retrieved using Salmon gene expression quantification data (MMRF_CoMMpass_IA15a_E74GTF_Salmon_V7.2_Filtered_Gene_TPM) in 774 BM_1 MM patients.

Clinical data regarding Overall Survival (OS) and Progression free Survival (PFS) were considered in 767 MM patients for which both RNA-seq expression and survival data were available.

Non-synonymous (NS) somatic mutation variants and counts data were obtained from whole exome sequencing (WES) analyses, main IgH translocations were inferred from RNA-seq spike expression estimates of known target genes and Copy Number Alteration (CNA) data were retrieved by means of Next generation Sequencing (NGS)-based fluorescence in situ hybridization (FISH) [1] in 497 MM cases for which all data were available [2].

The presence of a specific CNA was considered when occurring in at least one of the investigated cytoband at a 20 percent cut-off for each considered chromosomal aberration, as previously reported [2].

1.2. Survival analysis

Survival analyses were performed using survival [3,4] and survminer [5] packages in R Bioconductor (version 4.0.0). Kaplan-Meier analysis was applied on OS and PFS data in MIAT high/low expression groups according to maximally selected rank statistics, from the maxstat R package [6,7], using surv_cutpoint function in survminer package. A minimal proportion of 10% observable samples was set for each group and samples with lower or equal value as the cut-point were classified in low, greater that the cut-point in high group. Log-Rank test *p*-value was calculated to measure the global difference between survival curves. The number of samples at risk in each group across time was calculated. The global 767 MM dataset was half randomly split into train and test sets using createDataPartition function in caret [8] R package. According to this method, the random sampling occurs within each class and preserve the overall class distribution of MIAT expression level: 200 random splits were generated and the best cut-off associated with OS by max-stat method was chosen on train set and then validated on test set and on the global dataset.

The same procedure was applied to search for the best max-stat cutpoint of MIAT expression level possibly associated to PFS data.

The best cut-off that was identified in association to OS data was then tested with respect to OS on therapy groups in relation to bortezomib treatment.

Cox proportional hazards model was applied as univariate analysis on single molecular variables, age and International Staging System (ISS) groups in relation to OS data in 497 MM cases for which all information were accessible. Cox regression multivariate analysis was applied on all significant features after BH correction. Forest plot was used to summarize Cox Proportional Hazard Model.

1.3. Differential expression analysis

Global expression profiles of 19.141 protein-coding genes annotated by Ensembl Biomart were compared in fourth versus first MIAT quartile from the entire 774 MM RNA-seq dataset (194 MM cases in each group). Differentially expressed protein coding genes were selected by limma analysis, as previously described [2], at a False Discovery

Rate (FDR) < 10%. The same analysis was performed on the two extreme MIAT quartiles (128 MM cases in each group) in 512 MM RNA-seq dataset, after discarding 262 MM cases carrying t(11;14) or t(4;14).

1.4. Functional annotation enrichment analysis

Gene Set Enrichment Analysis (GSEA version 4.1.0) [9] was performed under default conditions on pre-ranked protein coding gene list based on fold-change values. Significant gene sets of Hallmark, Reactome, KEGG and C2-Gene Perturbations collections (version 7.2) were selected on the base of nominal p-value < 0.05. C2-Gene Perturbations gene sets were further filtered for Multiple Myeloma term.

Normalized Enrichment Score (NES) and False Discovery Rate (FDR) q-value are also reported.

References

1. Miller, C.; Yesil, J.; Derome, M.; Donnelly, A.; Marrian, J.; McBride, K.; Network, M.C.; Auclair, D.; Keats, J.J. A Comparison of Clinical FISH and Sequencing Based FISH Estimates in Multiple Myeloma: An Mmrf Commpass Analysis. *Blood* **2016**, *128*, 374–374, https://doi.org/10.1182/blood.v128.22.374.374.
2. Todoerti, K.; Ronchetti, D.; Favasuli, V.; Maura, F.; Morabito, F.; Bolli, N.; Taiana, E.; Neri, A. DIS3 mutations in multiple myeloma impact the transcriptional signature and clinical outcome. *Haematologica* **2021**, https://doi.org/10.3324/haematol.2021.278342.
3. Therneau T. A Package for Survival Analysis in R_. R package version 3.2-11, <URL: https://CRAN.R-project.org/package=survival> (accessed on 3 March 2021).
4. Therneau, T.M.; Grambsch, P.M. Modeling Survival Data: Extending the Cox Model. **2000**, https://doi.org/10.1007/978-1-4757-3294-8.
5. Alboukadel Kassambara, Marcin Kosinski and Przemyslaw Biecek. survminer: Drawing Survival Curves using 'ggplot2'. R package version 0.4.9. https://CRAN.R-project.org/package=survminer, (accessed on 3 March 2021).
6. Hothorn, T.; Lausen, B. On the exact distribution of maximally selected rank statistics. *Comput. Stat. Data Anal.* **2003**, *43*, 121–137, https://doi.org/10.1016/s0167-9473(02)00225-6.
7. Lausen, B.; Hothorn, T.; Bretz, F.; Schumacher, M. Assessment of Optimal Selected Prognostic Factors. *Biom. J.* **2004**, *46*, 364–374, https://doi.org/10.1002/bimj.200310030.
8. Max Kuhn.caret: Classification and Regression Training. R Package Version 6.0-86. Available online: https://CRAN.R-project.org/package=caret (accessed on 14 September 2021)
9. Subramanian, A.; Tamayo, P.; Mootha, V.K.; Mukherjee, S.; Ebert, B.L.; Gillette, M.A.; Paulovich, A.; Pomeroy, S.L.; Golub, T.R.; Lander, E.S.; et al. Gene set enrichment analysis: A knowledge-based approach for interpreting genome-wide expression profiles. *Proc. Natl. Acad. Sci.* **2005**, *102*, 15545–15550, https://doi.org/10.1073/pnas.0506580102.

Supplementary Figures

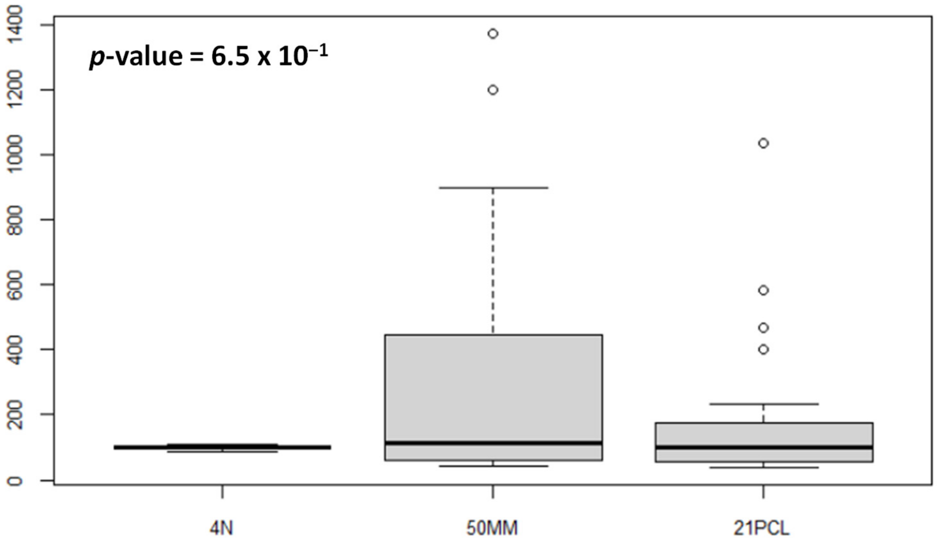

Figure S1. Box plots of MIAT expression level in healthy donors (N) and PC dyscrasias proprietary dataset. Total RNA samples from highly purified bone marrow CD138+ plasma cells were profiled by Gene 2.0 ST array. Kruskal-Wallis test was applied to assess differences in expression levels between groups.

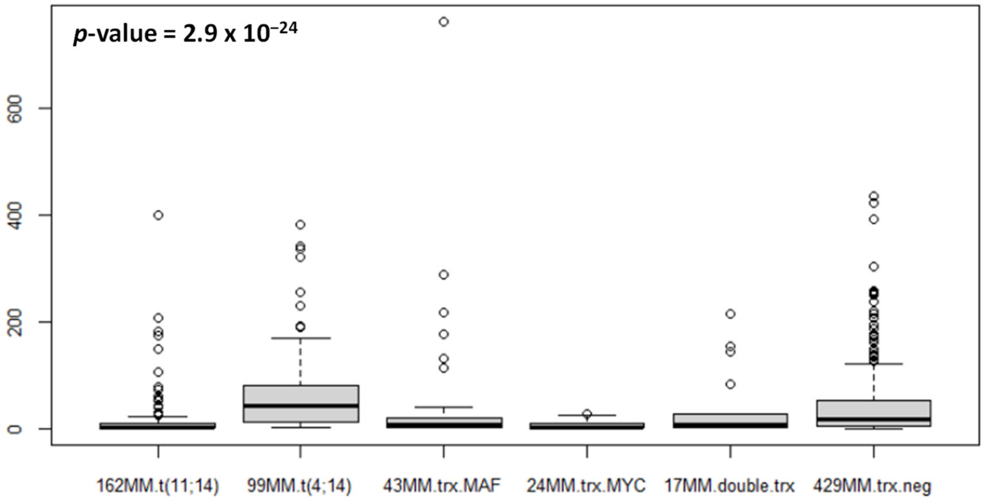

	t(11;14)	t(4;14)	Trx.MAF	Trx.MYC	Double trx
t(4;14)	**0.0000**				
Trx.MAF	**0.0047**	**0.0000**			
Trx.MYC	0.2576	**0.0000**	**0.0105**		
Double trx	**0.0489**	**0.0014**	0.4668	**0.0375**	
Trx neg	**0.0000**	**0.0000**	**0.0269**	**0.0000**	0.0895

Figure S2. Box plot of MIAT expression level in main IgH translocation groups in 774 cases of the CoMMpass cohort. Kruskal-Wallis test was applied to assess differences in expression levels between groups. The significant pairwise comparison performed by the Dunn test are marked red-bold in the table.

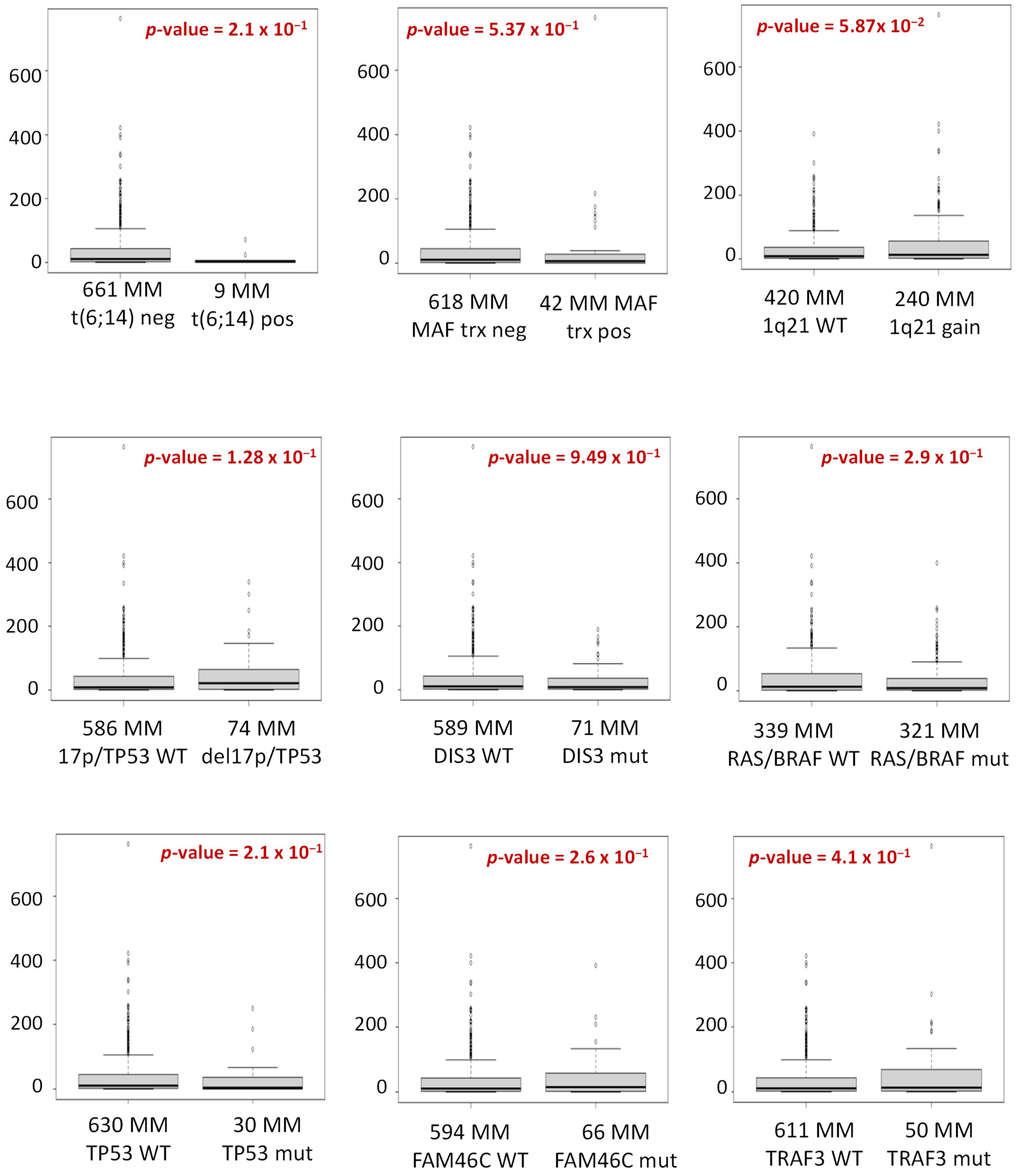

Figure S3. Box plots of MIAT expression level in 660 MM cases stratified according to the presence of t(6;14), MAF translocations, 1qgain, del(17p)/TP53 or the occurrence of NS somatic mutations in *DIS3*, *RAS*/*BRAF*, *TP53*, *FAM46C*, or *TRAF3* genes. Differential expression was tested by Wilcoxon rank-sum test with continuity correction. *p*-values were corrected by BH adjustment.

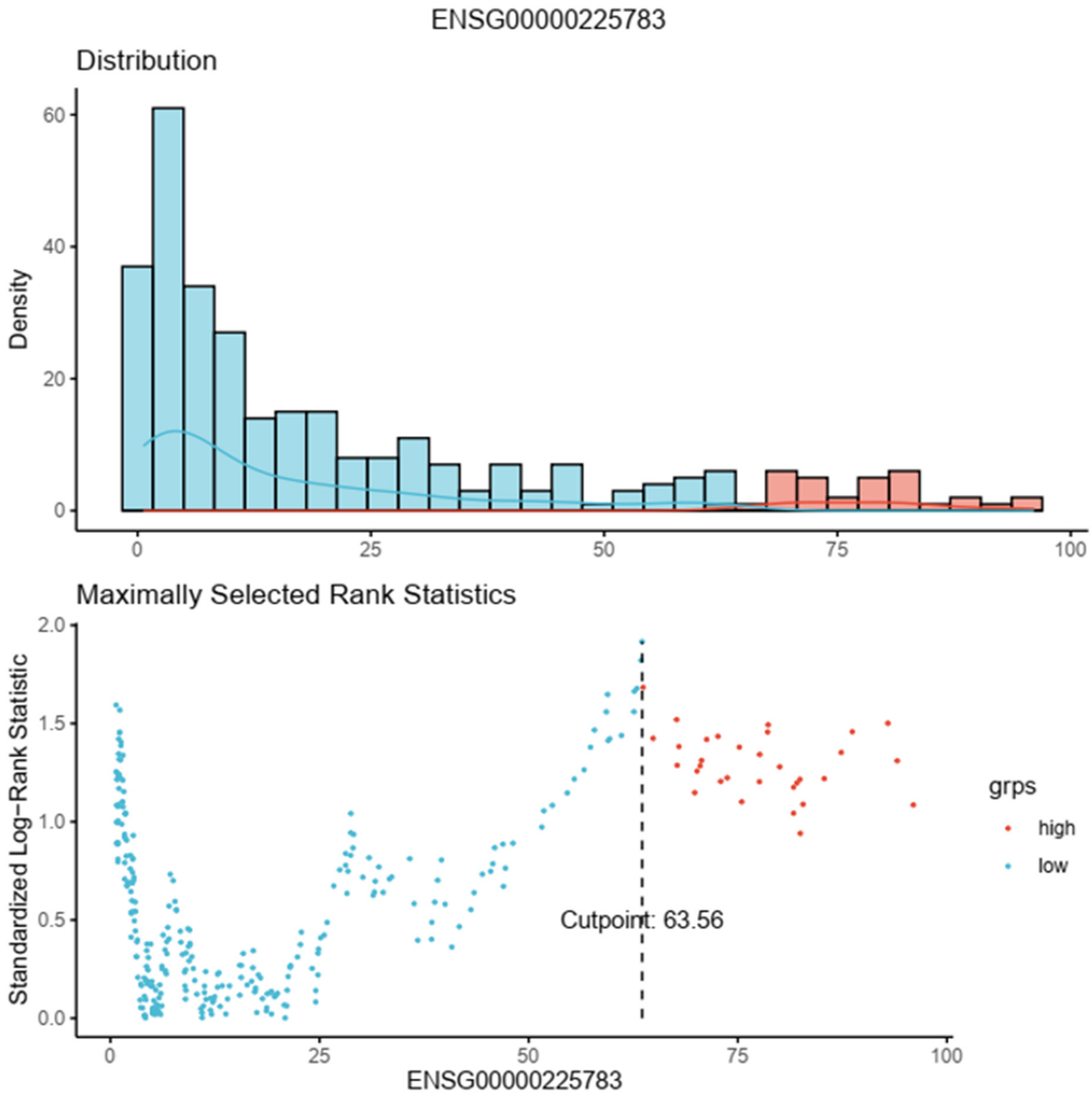

Figure S4. Determination of the optimal cut-point of MIAT expression level based on maximally selected rank statistics on 384 MM train set, randomly selected from 767 MM global dataset. A minimal proportion of 10% observable samples was set for each group. Sample with lower or equal value as cutpoint were classified in low, otherwise samples greater than cutpoint were classified in high group.

a

Variable	N	OS Univariate Cox Analysis		
		HR (95% CI)	P-value	Adj. P-value
high *MIAT*	92	1.74 (1.16-2.63)	0.00815	**0.02445***
older age (≥65 yrs)	206	2.12 (1.46-3.08)	8.68E-05	**5.00E-04*****
ISS I	187	0.31 (0.19-0.50)	1.86E-06	**0.00001953******
ISS II	171	1.13 (0.78-1.64)	0.524	0.68775
ISS III	139	2.45 (1.70-3.53)	1.62E-06	**0.00001953******
del(1p)/CDKN2C	143	1.61 (1.10-2.35)	0.0148	**0.034766667***
del(13q)/RB1	258	2.11 (1.44-3.09)	0.000119	**0.0004998*****
HD	281	0.64 (0.44-0.92)	0.0149	**0.034766667***
TP53.alterations	40	1.05 (0.53-2.07)	0.892	0.892
1q21 gain/amp	164	1.68 (1.17-2.43)	0.0051	**0.01785***
TP53.alterations + 1q21 gain/amp	19	3.63 (1.89-6.97)	0.000103	**0.0004998*****
DIS3 mut	50	1.63 (1.06-2.50)	0.0264	0.052690909
N-RAS mut	117	0.90 (0.62-1.32)	0.596	0.732666667
K-RAS mut	121	1.07 (0.77-1.49)	0.694	0.7623
BRAF mut	36	1.18 (0.60-2.31)	0.628	0.732666667
FAM46C mut	49	0.78 (0.42-1.43)	0.419	0.5866
TRAF3 mut	38	0.42 (0.17-1.06)	0.0668	0.107907692
t(11;14)	102	0.92 (0.58-1.46)	0.726	0.7623
t(4;14)	69	1.63 (1.04-2.55)	0.0335	0.058625
MAF.trx	33	1.96 (1.08-3.57)	0.0276	0.052690909
MYC.trx	20	1.92 (0.94-3.94)	0.0748	0.1122

b

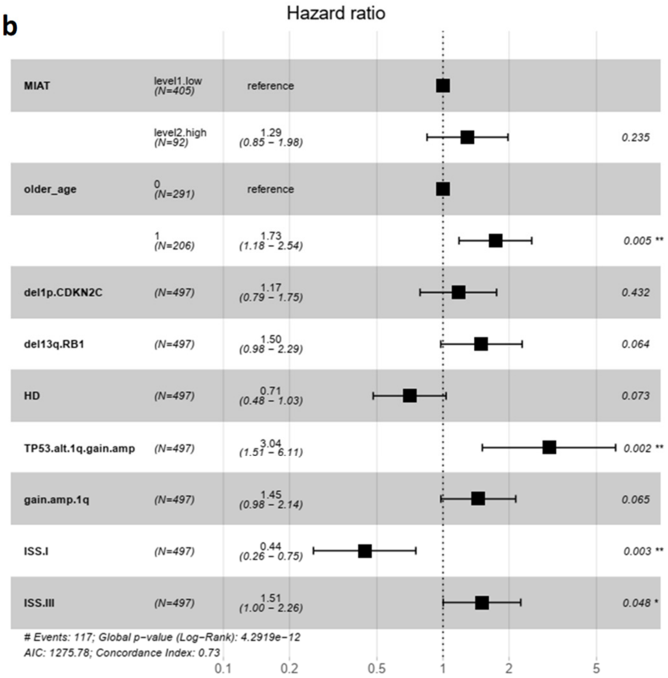

Figure S5. (**a**) Results of Cox regression univariate analysis using OS data on MIAT expression groups, age equal to or greater than 65 years, ISS subgroups and main molecular alterations in 497 BM-1 MM cases for which all data were available. Number (*N*) of positive cases is indicated for each variable. Hazard Ratio, 95% Confidence Interval and Log-rank *p*-value are reported for each variable. In red bold are depicted all significant variables after BH correction. (**b**) Forest plot of Cox regression multivariate analysis considering all features with adjusted *p*-value < 0.05 in univariate analysis with regards to OS in 497 BM-1 MM cases. Hazard Ratio, 95% Confidence Interval and Log-rank *p*-value are indicated in the plot for each variable. Significant *p*-value: * ≤ 0.05; ** ≤ 0.01; *** ≤ 0.001; **** ≤ 0.0001.

a

Variable	P-value	Pval_adj
high MIAT_del(13q)_RB1	0.000833	0.0029155
older age (≥65 yrs)	8.68E-05	5.00E-04
ISS I	1.86E-06	0.00001953
ISS II	0.524	0.68775
ISS III	1.62E-06	0.00001953
del(1p)/CDKN2C	0.0148	0.03476667
HD	0.0149	0.03476667
TP53.alterations	0.892	0.892
1q21 gain/amp	0.0051	0.0153
TP53.alterations + 1q21 gain/amp	0.000103	0.0004998
DIS3 mut	0.0264	0.05269091
N-RAS mut	0.596	0.73266667
K-RAS mut	0.694	0.7623
BRAF mut	0.628	0.73266667
FAM46C mut	0.419	0.5866
TRAF3 mut	0.0668	0.10790769
t(11;14)	0.726	0.7623
t(4;14)	0.0335	0.058625
MAF.trx	0.0276	0.05269091
MYC.trx	0.0748	0.1122

b

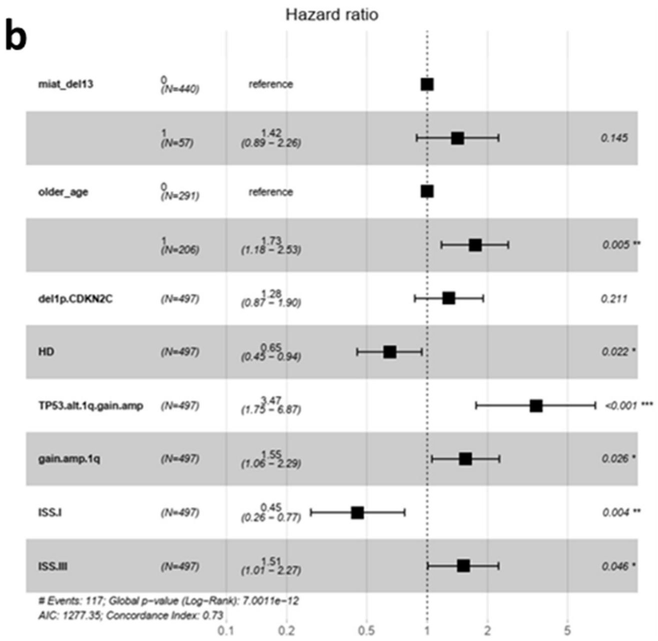

c

Variable	P-value	Pval_adj
high MIAT_1ploss	0.0152	0.03546667
older age (≥65 yrs)	8.68E-05	5.00E-04
ISS I	1.86E-06	0.00001953
ISS II	0.524	0.68775
ISS III	1.62E-06	0.00001953
del(13q)/RB1	0.000119	0.0004998
HD	0.0149	0.03546667
TP53.alterations	0.892	0.892
1q21 gain/amp	0.0051	0.01785
TP53.alterations + 1q21 gain/amp	0.000103	0.0004998
DIS3 mut	0.0264	0.05269091
N-RAS mut	0.596	0.73266667
K-RAS mut	0.694	0.7623
BRAF mut	0.628	0.73266667
FAM46C mut	0.419	0.5866
TRAF3 mut	0.0668	0.10790769
t(11;14)	0.726	0.7623
t(4;14)	0.0335	0.058625
MAF.trx	0.0276	0.05269091
MYC.trx	0.0748	0.1122

d

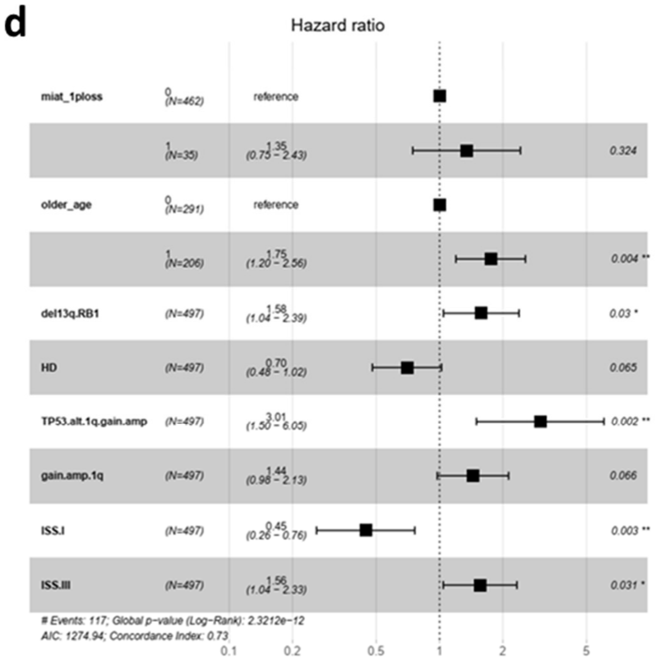

Figure S6. (**a**) Results of Cox regression univariate analysis using OS data on MIAT expression plus del13q groups, age ≥ 65 years, ISS subgroups and main molecular alterations in 497 BM-1 MM cases for which all data were available. (**b**) Forest plot of Cox regression multivariate analysis considering all features with adjusted *p*-value< 0.05 in univariate analysis with regards to OS in 497 BM-1 MM cases. (**c**) Results of Cox regression univariate analysis using OS data on MIAT expression plus del1p groups, age ≥ 65 years, ISS subgroups and main molecular alterations in 497 BM-1 MM cases for which all data were available. (**d**) Forest plot of Cox regression multivariate analysis considering all features with adjusted *p*-value < 0.05 in univariate analysis with regards to OS in 497 BM-1 MM cases. Hazard Ratio, 95% Confidence Interval and Log rank *p*-value are indicated in the plot for each variable. Significant *p*-value: * ≤ 0.05; ** ≤ 0.01;*** ≤ 0.001;**** ≤ 0.0001.

Therapy code	Therapy classification	680 total MM cases	130 high MIAT MM cases
1	combined bortezomib/IMIDs-based	356	71
2	Bortezomib-based	142	27
3	combined bortezomib/IMIDs/carfilzomib-based	25	2
4	combined IMIDs/carfilzomib-based	113	24
5	IMIDs-based	39	6
6	carfilzomib-based	5	0

Figure S7. Number of MM cases in each group of first-line therapy. Among 680 total MM cases with RNA-seq expression, clinical, and therapy data, the ones with high MIAT expression levels according to max-stat cut-off with respect to OS are also indicated.

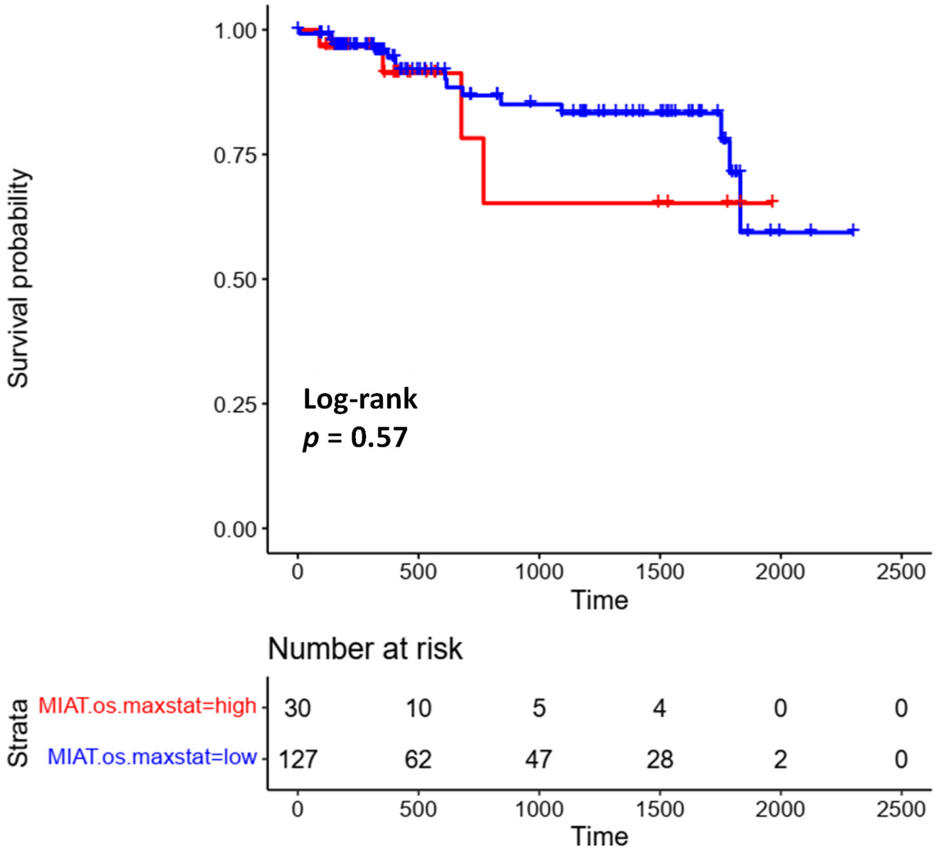

Figure S8. Kaplan-Meier survival curve in OS for 157 MM patients who underwent other regimens not including bortezomib. Samples were stratified in each dataset according to MIAT expression levels on the base of previous OS maxstat threshold. Log-rank test p-value measuring the global difference between survival curves and number of samples at risk in each group across time are reported.

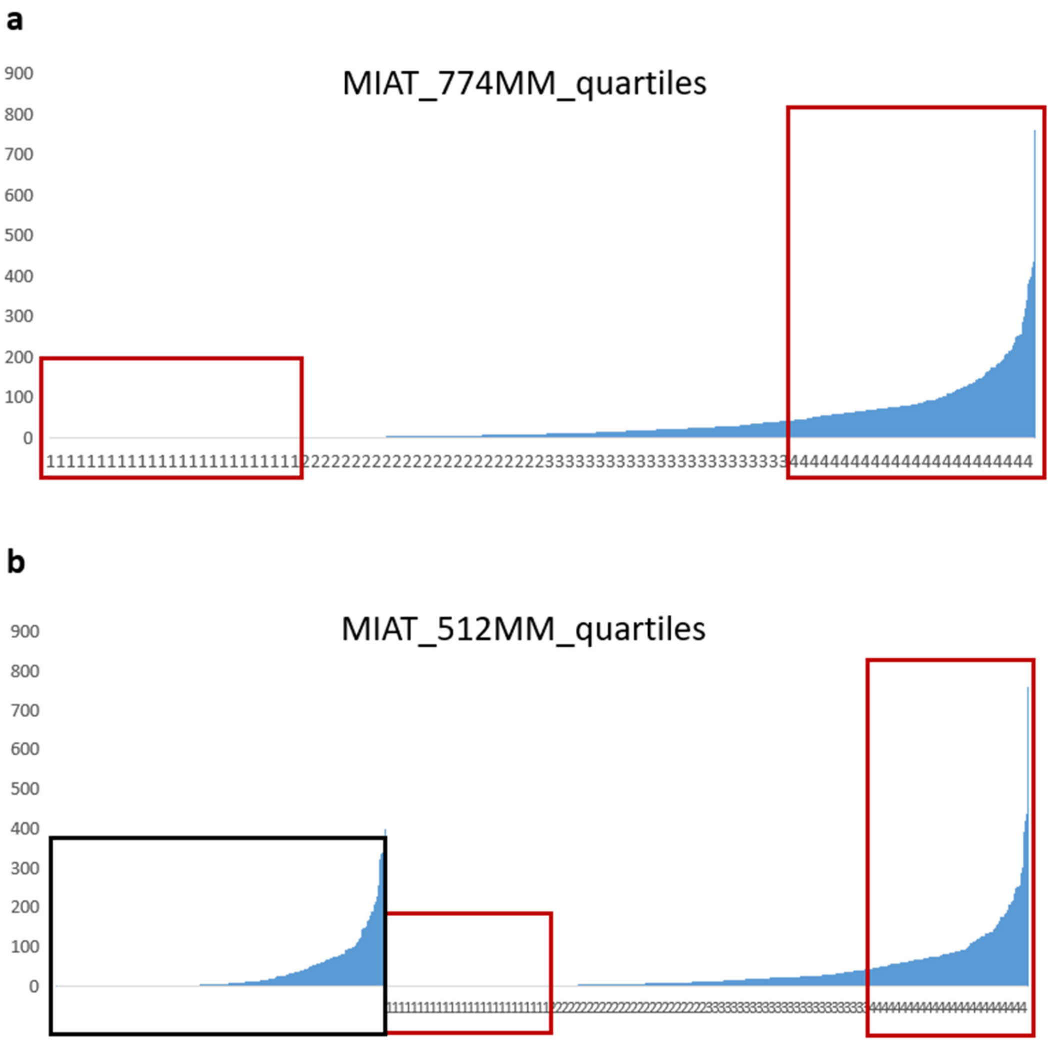

Figure S9. (**a**) MIAT expression levels in 774 MM of CoMMpass cohort according to quartiles. (**b**) MIAT expression levels in 512 MM dataset according to quartiles. Red boxes indicate the first and last quartiles in both datasets, the black one the discarded 262 MM cases carrying t(11;14) or t(4;14).

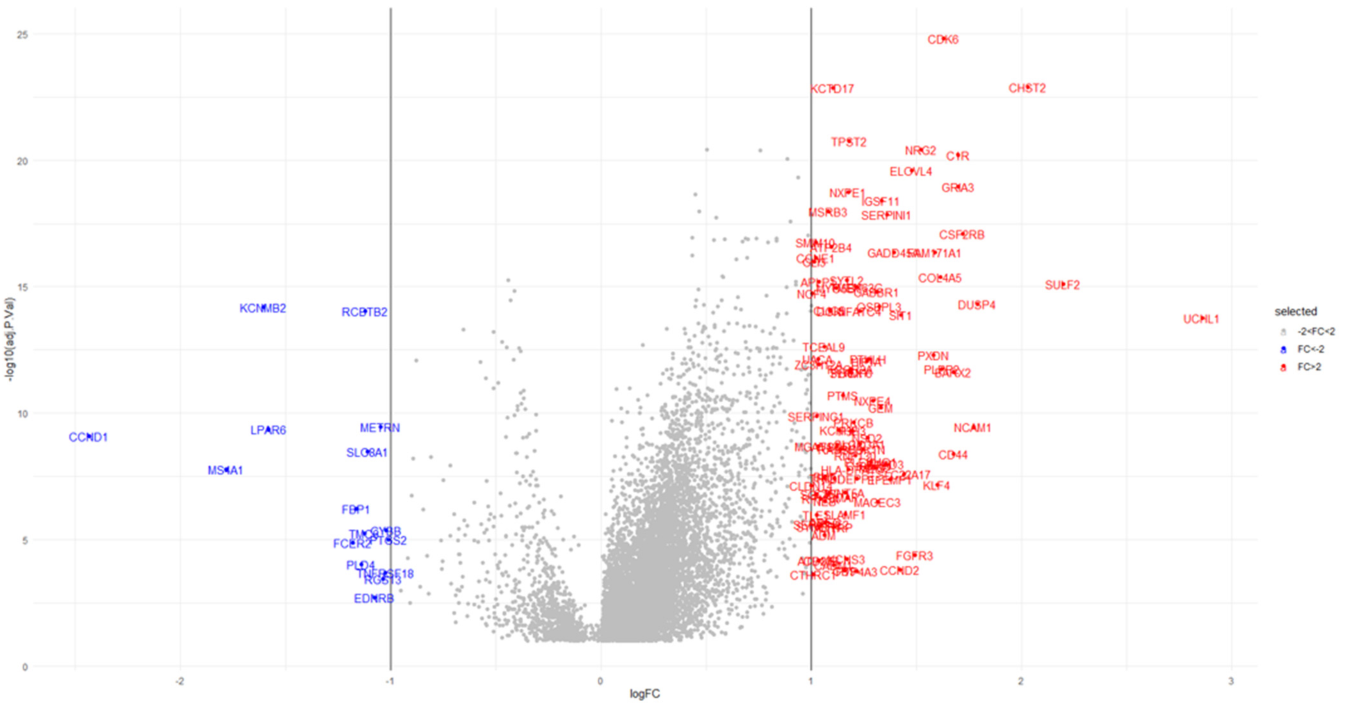

Figure S10. Volcano plot of 9813 significant DE protein coding transcripts between MIAT extreme quartiles. Up- and down-regulated transcripts with at least 2 FC absolute values are highlighted in red and blue, respectively.

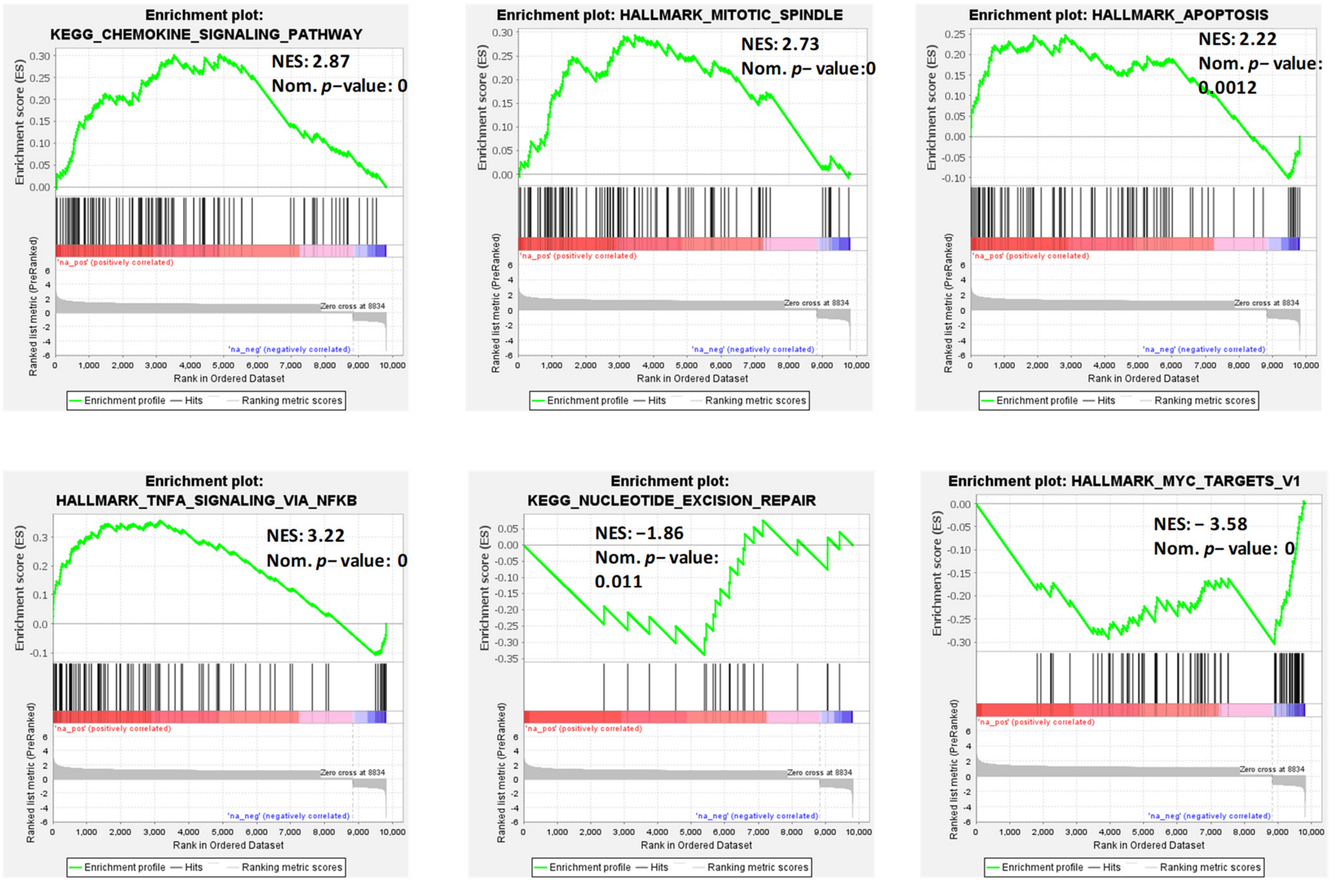

Figure S11. Enrichment plots of selected GSEA gene sets significantly up- and down-regulated in MIAT IV versus I quartile. Normalized Enrichment Score (NES) and nominal *p*-value are reported for each plot.
